# Gunshot Wounds in the Stomach—Successful Laparotomy

**Published:** 1885-01

**Authors:** 


### Gunshot Wounds in the Stomach—Successful Laparotomy.

Last month we gave the conclusions of Dr. Charles Parkes, of Chicago, as to the necessity of performing primary laparotomy in gunshot wounds of the abdomen. In this connection the following case, reported by Professor Kocher, of Beme, has considerable interest as tending to support the views of Dr. Parkes. A boy, aged 14, was admitted into hospital half an hour after receiving a wound in the region of the stomach, from a pistol shot aimed at him from a distance of about five paces.

He was pale and complained of abdominal pain; the abdomen was swollen and distinctly dull in percussion inferiorly. Pressure on the abdomen caused pain. A quarter of an hour later hiccough, severe epigastric pain, vomiting, pallor, and symptoms of collapse came on. There was tympanitic resonance from the ensiform cartilage to the umbilicus, with complete dullness from the navel downwards, and in the flanks; the lightest percussion caused severe pain. Three hours after the injury laparotomy was performed. On opening the abdominal cavity, in the region of the navel, a great quantity of dark blood escaped. The bullet wound was discovered with comparative ease; it was situated on the anterior surface of the stomach, towards the greater curvature, in the direction of the fundus. The wound was circular, with sharp edges, and about half an inch in diameter. The bullet could not be found, nor was there any aperture of exit. The edges of the wound were united, first with two catgut ligatures, like an ordinary wound, then a continuous silk ligature was applied, for the distance of about an inch, so as to invert the serous coat around the wound. Recovery was retarded by an abcess which formed in the track of the sutures in the abdominal wound. Professor Kocher declares that, considering the impossibility of recovery in cases of gunshot wounds of the stomach, when active measures are not taken, it is the duty of the surgeon to perform laparotomy whenever an injury of the kind is suspected.—*British Medical Journal, July* 12, 1884.

